# Semi-quantitative assessment of resting perfusion in chronic myocardial infarction

**DOI:** 10.1186/1532-429X-17-S1-P129

**Published:** 2015-02-03

**Authors:** Mita Patel, Victor Mor-Avi, Keigo Kawaji, Sandeep Nathan, Roberto Lang, Amit R Patel

**Affiliations:** Cardiology, University of Chicago, Chicago, IL USA

## Background

Based on observations from cardiovascular magnetic resonance (CMR) imaging, it is not clear whether chronic myocardial infarction (MI) is associated with abnormal perfusion at rest. Our aim was to investigate this question using semi-quantitative analysis of resting myocardial perfusion to compare areas of infarct and remote myocardium in patients with known coronary anatomy.

## Methods

We identified 19 patients who underwent regadenoson stress CMR (1.5T, Philips), had MI confirmed by late gadolinium enhancement (LGE), and underwent invasive coronary angiography within 6 months of CMR. Stress perfusion images were obtained during first pass of Gadolinium contrast agent one minute after regadenoson (0.4mg IV bolus) injection, and followed by reversal with aminophylline (75-125 mg), rest perfusion and late gadolinium enhancement (LGE) imaging 10-15 minutes later. Resting time-intensity curves were generated for a region of interest (ROI) in the area of MI, remote myocardium, and blood pool (Medis). Myocardial curves were used to obtain maximal up-slopes and normalized by the blood pool. Up-slopes were compared between the infarcted and remote myocardial ROIs.

## Results

There was no significant difference between the slopes in the infarcted and remote myocardium (0.31 ± 0.17 vs 0.32 ± 0.18 1/s) irrespective of presence of significant stenosis (>70%), or in ROIs supplied by arteries with or without significant stenosis (0.31 ± 0.18 vs 0.32 ± 0.17 1/s) irrespective of presence of scar.

## Conclusions

Resting myocardial perfusion on CMR images does not reflect either the presence of chronic MI or underlying coronary patency. Accordingly, normal resting perfusion should not be used to rule out either significant stenosis or the presence of an underlying MI.

## Funding

N/A.

**Figure 1:**
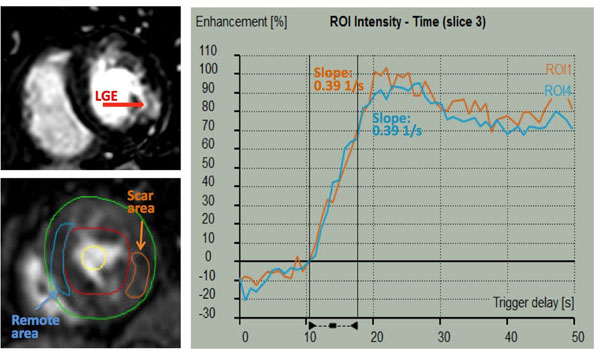
Time intensity curves for infarcted and remote myocardium at rest.

